# Organoids and Liquid Biopsy in Oral Cancer Research

**DOI:** 10.3390/jcm9113701

**Published:** 2020-11-18

**Authors:** Takanori Eguchi

**Affiliations:** 1 Department of Dental Pharmacology, Graduate School of Medicine, Dentistry and Pharmaceutical Sciences, Okayama University, Okayama 700-8525, Japan; eguchi@okayama-u.ac.jp; 2 Advanced Research Center for Oral and Craniofacial Sciences (ARCOCS), Okayama University Graduate School of Medicine, Dentistry and Pharmaceutical Sciences, Okayama University, Okayama 700-8525, Japan; 3 Department of Dental Pharmacology, Okayama University Dental School, Okayama 700-8525, Japan

## 1. A Special Issue of Oral Cancers

To promote the newest discoveries in oral cancer research, a special issue “Frontiers in Oral Cancer—Basic and Clinical Sciences” in the Journal of Clinical Medicine (JCM) was opened from September 2019 to April 2020. A total of 8 papers were published, including a single review and seven original articles. Firstly, “Organoids and Tumoroids” are growing approaches in oncology [1,2] and are reviewed in this issue [3]. Secondly, “Saliva Biopsy” is a minimally invasive liquid biopsy compatible with oral and systemic status. In this issue, saliva biopsies were attempted by three groups to discover novel, veridical biomarkers. Besides, extracellular vesicles (EVs) and biomarkers in saliva are becoming crucial [4,5]. Thirdly, genomic and epigenomic alterations are essential in cancer research [6,7]. In this issue, these are examined by The Cancer Genome Atlas (TCGA)-based informatics and next-generation sequencing (NGS) of methyl-DNA, respectively. Finally, novel mechanisms of drug resistance are being discovered [8]. This issue includes two papers featuring drug resistance in oral cancer. 

Oral cancers arise from oral mucosa and most are histologically squamous cell carcinomas. Oral squamous cell carcinoma (OSCC) is included in the head and neck squamous cell carcinoma (HNSCC) category, which has different risk factors depending on the site of occurrence. The most significant risk factors for oral cancers are habitual tobacco use and smoking, which operate synergistically with drinking alcohol. In particular, human papillomavirus (HPV) is a recognized etiological factor in oropharyngeal cancer, but it only occurs with a small minority of OSCCs. Oral cancers and leukoplakia, one of oral potentially malignant disorders, are exposed to the oral cavity and thus easily accessible for diagnoses and therapies. Early detection and early treatment of oral cancers are each correlated with a good prognosis while delayed detection is correlated with poor prognosis involving metastasis to neck lymph nodes, lungs, and other organs. Surgery, chemotherapy, radiation therapy, and their combinations are currently standards of care (SOC) while immunotherapies, super-selective intra-arterial chemotherapy, and photo-immunotherapy have been recently applied. However, therapy resistance and metastasis remain unsolved issues. Veridical biomarkers for early detection, precise diagnosis, and reliable prognosis are also required. Tumoroids, saliva biopsies, genomics, and epigenomics are currently key approaches to overcome these issues.

## 2. Tumoroids and Organoids

Tumoroids a.k.a. tumor organoids were reviewed by Tatullo et al. with 77 references [3]. The scientific research on cancer biology was based on in vitro experiments, carried out on tissue culture plates and two-dimensional (2D) samples. In this context, the formation of colonies, three-dimensional (3D) tumorspheres and spheroids have been found as morphological markers of malignancy and stemness of tumor cells [2,9]. More recently, 3D printing-based 3D culture systems have greatly improved in-vitro tumor models based on new biological matrices that mimic the extracellular environments. Indeed, organoids have been described in many reports on scientific literature. The term that better describes such organoids-based tumoral tissues is “tumoroids”. Tatullo et al. updated the review of tumoroids applied in translational medicine, paying specific attention to their use in the investigation of the main molecular mechanisms of cancer onset and growth and the most impactful strategies for effective targeted therapies. Besides, biomaterials such as matrigel have been often used for organoid/tumoroid studies while gel-free, liquid-based tumoroid (LBT) system [10] enables easy isolation and addition of cells, drugs [2], and vesicles [1,11] in the culture media. The LBT systems also enable high-throughput (HTP) screenings [12]. 

## 3. Liquid Biopsy

Novel biomarkers are being found in liquid biopsies of saliva or their EVs. Saliva enables a non-invasive liquid biopsy, is easily accessible, and is plentiful in resources of biomarkers for diseases as well as pre-symptomatic and health status. Because oral cancer and potentially malignant disorders are physically exposed to an oral cavity, salivary biomarkers may be useful for their early detection. 

In this issue, three groups featured liquid biopsies to seek salivary, plasma, and urine biomarkers, including mRNA biomarkers, metabolites, and pro-inflammatory cytokines in OSCC. Oh et al. reported salivary mRNA biomarkers for early detection of oral cancers [13]. They selected 30 candidate genes relevant to cancer from recent reports. Saliva samples were obtained from more than 30 OSCC patients and non-tumor controls. The mRNA levels of six genes (NAB2, CYP27A1, nuclear pore complex interacting protein B4 (NPIPB4), monoamine oxidase B (MAOB), sialic acid acetyltransferase (SIAE), and collagen type III alpha 1 (COL3A1)) were significantly “lower” in the saliva of OSCC patients. The combination of CYP27A1 and SIAE showed a favorable area under the curve (AUC) value of 0.84. The AUC of MAOB–NAB2 was more predictive of OSCC in the under-60 group (AUC, 0.91; sensitivity, 0.92; and specificity, 0.86) than any other combination of the transcripts. These studies may aid the early diagnosis of OSCC, especially in patients under 60 years of age, and suggest salivary mRNAs to be potent biomarkers for early OSCC diagnosis.

Next, pro-inflammatory, NF-κB-dependent cytokines (IL-1α, IL-6, IL-8, and TNF-α) were evaluated in saliva and tissue specimens of patients with OSCC and oral potentially malignant disorders [14]. Salivary, NF-κB-dependent pro-inflammatory cytokines and matrix metalloproteinases (MMPs) may link oral cancers with aging involving the senescence-associated secretory phenotype (SASP), inflammatory diseases such as periodontitis, and wound in oral mucosas [15]. 

Metabolome biomarkers in OSCC were also examined to identify high-risk patients with extranodal extension by using Nuclear Magnetic Resonance (NMR) metabolomics [16].

Besides this, recent studies have disclosed EVs, including exosomes (small vesicles) and larger vesicles, released from oral tissues and/or oral cancer lesions into saliva [4]. Many protein biomarkers of oral cancers have been found from EVs, including epidermal growth factor receptor (EGFR) [17] and heat shock proteins (HSPs) [18,19,20]. Salivary microRNAs in EVs may be also key molecules for early diagnosis of oral cancers distinguished from periodontitis [5], aging [21], and health status [22].

## 4. Genome and Epigenome Alterations

Genetic and epigenetic alterations are essential changes found in many cancers. DNA hypermethylation in tumor suppressor genes (TSG) and functions of tumor-suppressive microRNAs (TS-miR) [6,7] are associated with lymph node metastasis in OSCC as well as esophageal squamous cell carcinomas (ESCC) [23,24]. In this issue, Gissi et al. revealed methyl-DNA in 13 genes from “oral brushing” in the follow-up of oral cancer patients [25]. Quantitative methyl-DNA analysis was performed by using NGS for ZAP70, ITGA4 (α4 integrin), KIF1A, PARP15, EPHX3, NTM, LRRTM1, FLI1, MIR193 (miR-193), LINC00599 (long intergenic non-coding RNA: lincRNA), MIR296 (miR-296), TERT (telomerase reverse transcriptase), and GP1BB genes. In addition to the salivary biopsy approach described above, oral brushing is now a non-invasive, promising tool for biopsy in oral cancers.

In addition, the genomic landscape in HNSCC was disclosed by analyzing the TCGA database [26]. Sayans et al. comprehensively described somatic DNA alterations and transcriptional alterations in the last extension of the HNSCC subsets in TCGA, encompassing a total of 528 tumors. Somatic copy number alteration analysis revealed the top-five altered genes in HNSCC: deletions of cyclin-dependent kinase (CDK) inhibitory genes (CDKN2A and CDKN2B) followed by amplifications of PPFIA1, FADD, and ANO1. Survival analysis revealed that overexpression of oncogenes such as EGFR and CDK4/6 was associated with poorer prognosis. These comprehensive findings may help us to develop new therapies and programs for the prevention of HNSCC.

## 5. Drug Resistance

In accordance with excessive CDKs in HNSCC disclosed by the genomic studies, Remer et al. reported that CDK4/6 inhibitors are effective to overcome acquired, inherent resistance to PI3Kα inhibition in pre-clinical models of HNSCC [27]. Besides, a CDK2 inhibitor could be another approach to target cancer drug resistance [12]. Moreover, a vesicle-based drug resistance mechanism has been recently discovered [8,28]. 

Shimomura et al. reported that NCAPH is associated with lymphangiogenesis and drug resistance in OSCC. Lymphangiogenesis is nowadays regarded as being crucial in the tumor microenvironment, tumor immunology, and metastasis, in addition to tumor angiogenesis [29]. 

## 6. Summary

Taken together, (1) tumoroids and organoids are growing approaches in oral cancer research; (2) salivary biopsy is beneficial for the discovery of novel, veridical biomarkers; (3) genomic and epigenomic alterations are reliable biomarkers, including methyl-DNA, miRNA, and lincRNA; and (4) multiple, novel mechanisms of drug resistance are being disclosed in oral cancers.
